# Mild hypothermia of 34°C reduces side effects of rt-PA treatment after thromboembolic stroke in rats

**DOI:** 10.1186/2040-7378-4-3

**Published:** 2012-03-07

**Authors:** Bernd Kallmünzer, Stefan Schwab, Rainer Kollmar

**Affiliations:** 1 Department of Neurology, University of Erlangen, Germany

**Keywords:** focal ischemia, stroke, thrombolysis, hypothermia, reperfusion, MRI, thromboembolic model, rat

## Abstract

**Background:**

Hypothermia is neuroprotective in experimental stroke and may extend the so far limited therapeutic time window for thrombolysis. Therefore, hypothermia of 34°C and its effects on delayed thrombolysis including reperfusion-associated injury were investigated in a model of thromboembolic stroke (TE).

**Methods:**

Male Wistar rats (n = 48) were subjected to TE. The following treatment groups were investigated: control group - normothermia (37°C); thrombolysis group - rt-PA 90 min after TE; hypothermia by 34°C applied 1.5 to 5 hours after TE; combination therapy- hypothermia and rt-PA. After 24 hours infarct size, brain edema and neuroscore were assessed. Protein markers for inflammation and adhesion, gelatinase activity, and blood brain barrier (BBB) disruption were determined. MRI-measurements investigated infarct evolution and blood flow parameters.

**Results:**

The infarct volume and brain swelling were smaller in the hypothermia group compared to the other groups (p < 0.05 to p < 0.01). Thrombolysis resulted in larger infarct and brain swelling than all others. Hypothermia in combination with thrombolysis reduced these parameters compared to thrombolysis (p < 0.05). Moreover, the neuroscore improved in the hypothermia group compared to control and thrombolysis. Animals of the combination therapy performed better than after thrombolysis alone (p < 0.05). Lower serum concentration of sICAM-1, and TIMP-1 were shown for hypothermia and combination therapy. Gelatinase activity was decreased by hypothermia in both groups.

**Conclusions:**

Therapeutic hypothermia reduced side-effects of rt-PA associated treatment and reperfusion in our model of TE.

## Introduction

Thrombolysis by recombinant tissue-plasminogen activator (rt-PA) is the preferable causal therapy for acute ischemic stroke, but only a minority of all stroke patients is eligible for treatment [1]. Its approval is restricted to the first 4.5 hours after symptom onset [2,3]. Delayed administration of rt-PA has less pronounced effects on restoration of cerebral blood flow (CBF) and outcome, but may still be effective [2-4]. However, clinical and animal data suggest an increased risk for intracerebral hemorrhage and brain edema after delayed thrombolysis [4,5]. Possibly, these side effects account to a reperfusion-associated injury [6], pro-apoptotic and neurotoxic side effects of rt-PA [7,8] with dysregulation of Matrix Metalloproteinases (MMPs) and disruption of the blood brain barrier (BBB) [9].

Hypothermia might be a promising candidate for combination therapy with rt-PA because of its multiple neuroprotective effects and capacity to reduce reperfusion associated injury [10,11]. Moreover, it is the only strategy that succeeded in acute brain injury so far: moderate hypothermia (33°C) improved functional outcome and survival of cardiac arrest patients when applied directly after successful resuscitation [12]. New approaches to counteract cold-induced shivering and patient discomfort allow mild hypothermia to be administered in awake stroke patients [13]. Moreover, first results from clinical trials suggest that the combination of rt-PA and hypothermia might be feasible and safe [14,15].

In this study, we investigated the effects of induced hypothermia on rt-PA related reperfusion damage in a model of thromboembolic stroke (TE). Hypothermia of 34°C was used as it was the most effective target temperature in a previous dose escalating study after filament occlusion of the middle cerebral artery [16]. Infarct size and brain swelling were investigated by silver infarct staining and magnetic resonance imaging (MRI). Matrix metalloproteinases-1 (TIMP-1), and soluble intercellular adhesion molecule (sICAM-1) were measured as markers of reperfusion- and rt-PA-associated injury.

## Materials and methods

### Experimental procedure

Animal experiments were approved by the local ethics committee. Male Wistar rats (n = 112) weighing 280-320 g (Charles River, Sulzfeld, Germany) were subjected to embolic stroke (TE) using a method modified from Busch [17].

#### Preliminary study

In a **preliminary study **(n = 64 animals), the effects of different red blood clot size and its various preparation on infarct size, edema and mortality have been tested in four different groups (n = 16 each). Anaesthesia and animal preparation were performed as described for the main body of the study. No rt-PA has been given.

A whole blood sample of 0.5 ml was withdrawn into a polyethylene catheter (PE-50; NeoLab, Heidelberg, Germany) and allowed to clot for 2 hours. Then, blood clots were transferred to a 20 mM CaCl_2 _solution and incubated over night at 4°C or for several minutes at room temperature. Prior to cutting clots the calcified blood was washed in 0.9% NaCl to wash out the CaCl_2_. A blood clot of 30 mm or 24 mm length, respectively, was divided into 12 pieces on a scale paper. Clot preparation according to treatment groups has been done as follows:

*Group 1: *Clot incubation time 24 hours, clot size 3 mm, number of clots 12

*Group 2: *Clot incubation time 2 hours, clot size 3 mm, number of clots 12

*Group 3: *Clot incubation time 24 hours, clot size 2 mm, number of clots 12

*Group 4: *Clot incubation time 2 hours, clot size Group 2 mm, number of clots 12

After 24 hours, the animals have been sacrificed by an overdose of ketamine (10%) and xylazine hydrochloride (100 mg/kg body weight). After decapitation, brains were rapidly removed and dissected into 5 coronal sections of 2 mm thickness, incubated in a 2% solution of 2,3,5-triphenyltetrazolium chloride (TTC) at 37°C for 15 minutes until the brains were stained sufficiently and immersion-fixed in a 4% paraformaldehyde solution. Slices were scanned into Adobe Photoshop and infarct size was measured using scion image (Scion corporation, Maryland, USA) as imaging software. Infarct size is reported as infarct volume and is corrected for edema. To compensate for the effect of brain edema, the corrected infarct volume was calculated as previous described [16]. The following formula was used to calculate the infarct volume corrected for the cerebral edema [16]: Corrected infarct volume = volume of the left, nonischemic hemisphere - (volume of the right, ischemic hemisphere - infarct volume). The volume of the brain edema has been calculated with the following formula: the sum of surface areas of the left, non-ischemic hemisphere divided by the sum of surface areas of the right, ischemic hemisphere. Infarct size and size of edema is expressed as % of the contralateral hemisphere.

Since mortality was the lowest in group 4 of the preliminary study and infarct size showed low standard deviations, clots for animals in the major study were prepared according to method 4.

#### Main study

Animals were randomly assigned to one of the following groups:

*Control group (Co): *37°C body core temperature, no specific treatment (n = 12).

*Thrombolysis group (T): *37°C body core temperature, rt-PA 1.5 h after TE (n = 12).

*Hypothermia group (H): *34°C induced 1.5 h after TE and maintained for 4.5 h (n = 12).

*Combination group (TH): *34°C induced 1.5 h after TE and maintained for 4.5 h plus rt-PA 1.5 h after TE (n = 12).

##### General anaesthesia and monitoring of vital signs

Animals were anesthetized using a mixture of halothane (Halocarbon laboratories), oxygen 30% and N_2_O (70%). Minimum alveolar concentrations were corrected for the actual body temperature. A polyethylene-catheter (PE-50; Neolab, Heidelberg, Germany) in the right femoral artery was used for continuous monitoring of blood pressure, heart frequency, and blood gases during the experiment as well as blood sampling for measuring blood protein levels. The right femoral vein was cannulated for administration of rt-PA, placebo or contrast agent (Magnevist).

##### Thromboembolic stroke model

For induction of TE, the right common carotid (CCA), internal carotid (ICA), and external carotid arteries (ECA) were exposed, and further dissection identified the origin of the pterygopalatine artery (PPA). The ECA and the PPA were permanently ligated, while the CCA was only temporarily clipped for embolization. A PE 50 catheter was inserted into the ECA proximal to its ligation, and 12 red blood clots (each 0.35 mm in diameter and 2 mm in length) were injected at the origin of the right middle cerebral artery (MCA).

##### Temperature management

Rectal temperature was regulated by a thermostatically controlled heating pad (Foehr Medical Instruments, Seeheim-Jugenheim, Germany). A rectal probe was inserted 4 cm into the rectum to measure the actual body core temperature. Prior experimental data indicated that the body core temperature correlates to intracranial and pericranial temperature during normothermia and therapeutic hypothermia [18]. In normothermic rats, a target rectal temperature of 37°C was achieved by setting the heating pad to 37°C. For induction of therapeutic hypothermia, the animals were externally cooled by cooling pads attached to the abdomen of the animal in the prone position, until a temperature of 34.5°C was reached. Thereafter, cooling pads were removed from abdomen and the heating pad was set to 34°C. Within approximately 2-5 min, the animal reached 34°C which was maintained via the heating pad for the entire cooling period. The Optimal temperature of 34°C of body temperature for effective treatment of experimental stroke has been described in our laboratory and cooling of the animals was performed as described in this previous study [16]. Rewarming was performed by setting the heating pad to the desired target temperature of 37°C. The rewarming procedure took approximately 30 min.

##### Thrombolytic treatment

For thrombolysis, rt-PA (Boehringer Ingelheim, Germany) 10 mg/kg body weight was injected 1.5 hours after induction of TE as previously described [19].

##### Removal of brain tissue

Twenty-four hours after MCAO, rats were sacrificed by an overdose of ketamine (10%) and xylazine hydrochloride (100 mg/kg body weight). After decapitation, brains were rapidly removed, frozen in isopentane at -20°C, and stored until use at -80°C.

### Infarct size and brain edema

The silver infarct staining (SIS) method was used to evaluate the infarct size. With this method, ischemic brain tissue can be reliably distinguished from nonischemic white and gray matter of rat brain cryosections as soon as 2 hours after MCAO [20]. Frozen brains were dissected into 14-μm sections on 5 coronal levels 2 mm apart from each other. A semiautomated method was used to measure the cerebral infarct volume and brain swelling as described previously [19]. The brain sections were analyzed by an investigator who was blinded for the treatment of the groups. All reported data for infarct volume are corrected for edema.

### Neuroscore

24 hours after TE, all animals were tested for neurological outcome using the neuroscore according to Menzies [21]: 0 = no apparent deficit, 1 = contralateral forelimb flexion; 2 = decreased grip of contralateral forelimb grip while tail pulled; 3 = spontaneous movement in all directions, contralateral circling only if pulled by tail; 4 = spontaneous contralateral circling. The testing was performed by a co-worker who was blinded for the earlier treatment regimen.

### Immunohistochemistry

Frozen sections of 14 μm thickness out of the level -1.4 to -1.8 mm to bregma were used for immunohistochemical analysis. Studies were performed with antisera against myeloperoxidase (MPO; DAKO) and performed according to the manufacturer's advice.

MPO-positive cells were quantitatively measured counting MPO positive cells per infarcted and non-infarcted hemisphere of all groups [16].

### *In situ *Zymography

Frozen cryostat brain sections of ischemic rats (n = 4 per group) were brought to room temperature (RT) and incubated with 100 μl FITC-labeled DQ-gelatin (Invitrogen, Eugene, USA) overnight at 37°C in a humidified chamber. These sections had a 14 μm thickness out of the level -1.4 to -1.8 mm to bregma. The sections were washed with PBS, fixed in 1% Paraformaldehyde and examined by fluorescence microscopy to reveal gelatinase activity at the cellular level. Sections were also stained with a monoclonal antibody (mAb) against neuronal nuclei (NeuN, Chemicon, USA; diluted 1:500) followed by a Cy3-labeled secondary Antibody (Zymed Laboratories, San Francisco, USA). 3 regions of interest (ROI) were investigated: parietal Cortex, hippocampus, basal ganglia. A qualitative assessment was performed. A quantitative measurement of fluorescence activity according to gelatinase activity was done and values were expressed as relative increase of fluorescence in the ischemic hemisphere in contrast to the non-ischemic hemisphere.

### Marker-proteins

Arterial blood was drawn at 4 time points to measure protein values of TIMP-1, and sICAM-1: Prior to TE, 0.5 hours after TE, 4 hours after TE, and 24 hours after TE. Commercial ELISA Kits were used to determine different protein concentrations (Quantikine rat TIMP-1; Quantikine rat sICAM-1; all R&D Systems, Wiesbaden, Germany).

### Magnetic resonance imaging (MRI) protocol

Animals were subjected to MRI at three time points: 0.5 hours after TE (DWI, PWI); 4 hours after TE (T2, PWI); 24 hours after TE (T2, PWI).

The animals were examined in a 2.35-T scanner (Biospec 24/40, BRUKER Medizintechnik, Ettlingen, Germany), using an actively shielded gradient coil with an inner diameter. The coil operates on a standard 150 V/100 2 gradient power supply. In this configuration, 180 mT/m could be reached in 180 ms. AsRF coil, we used a home-built birdcage resonator with an inner diameter of 40 mm. At each time point, MRI data were acquired for 20 min. MRI sequences are described in detail previously [19]. No infarcts were observed outside the imaged regions. A blinded investigator measured the lesion volumes of the T2- and DWI-weighted MR images by tracing the area of hyperintense regions. All reported data for infarct volume are corrected for edema. From the PWI data, we calculated the relative regional cerebral blood volume (rrCBV) as described previously [22] only after TE to assess valid occlusion of the MCA. PWI data were assessed as region of interest (ROI) with an area of 3 × 3 pixels at the level of the lateral basal ganglia, which are included in the ischemic area in the used model. Values were then calculated as a percentage of the non-ischemic hemisphere.

### Statistical analysis

Parametric data such as infarct size and weight were compared by analysis of variance (ANOVA) and Scheffé post hoc tests if needed. Physiological data were compared by 2-way ANOVA with 3 within time points. Fisher's protected least significant difference (PLSD) correction confirmed significant group effects. The nonparametric Kruskall-Wallis test evaluated the neuroscore with subsequent group comparisons by Mann-Whitney *U *test. The Chi square test with Yates correction for small numbers was used to test for differences in hemorrhagic transformation and mortality rate. sICAM and TIMP-1 values were assessed by repeated measures ANOVA, since blood draws were taken over time.

An alpha error rate of 0.05 was taken as the criterion for significance for all tests. Data are presented as mean ± SD if not stated otherwise. Analyses were performed with StatView^® ^statistical software (SAS Institute Inc., 1998).

## Results

### Preliminary study

**Figure 1:**
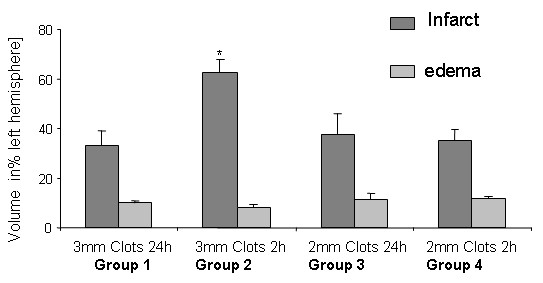
**Infarct size (dark grey bars), and brain swelling (light grey bars) are shown according to clot preparation after 24 hours as a percentage of the left hemisphere and are presented as mean ± SD**. Infarct size is significantly higher compared to all others in group 2 (p < 0.01; asterisks).

The mortality rate was the highest in group 2. 10 animals died before reaching 24 hours. 7 of 16 animals died in group 1, 9 of 16 animals died in group 2, 6 of 16 animals died in group 3 and only one animal died in group 4. There was not difference in the infarct size between group 1, 3 and 4. However, infarct size was significantly higher in group 2 compared to all other (p < 0.05). Edema did not differ between the groups (Figure 1).

### Main Study

#### Physiological parameters

There was no significant difference for the measured physiological parameters with the exception of the desired body temperature according to the treatment group (data not shown). The body temperature was in the desired range during the experimental period and during sedation. Levels were 37 ± 0.2°C during normothermia in all groups and 34 ± 0.2°C during the hypothermia period. There were no significant differences within the normo- and hypothermic periods.

#### Mortality

2 animals of the control group died within 24 hours after TE. All other groups exhibited 3 dead animals during the same time period. Differences were not statistically significant.

#### Infarct size and brain swelling in silver infarct staining

**Figure 2:**
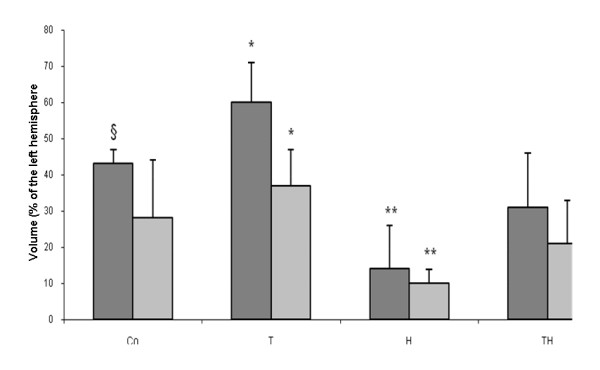
**shows results of the main study**. Infarct size and brain swelling in group T are significantly larger than in all other groups (p < 0.01; asterisks). Infarct size in group Co is larger than in group TH (p < 0.05; paragraph sign). Infarct size and brain swelling are smaller in group H compared to group Co (p < 0.01) and TH (p < 0.05), double asterisks.

Infarct volume were calculated as follows: group Co 140 ± 80 mm^3^, group T 278 ± 102 mm^3^, group H 75 ± 57 mm^3^, and group TH 120 ± 42 mm^3^. The infarct volume in group T was significantly larger compared to all other groups (p < 0.01). Group H showed a significantly lower infarct size in comparison to group Co and TH (p < 0.05). Differences in brain swelling accounted to the same significant differences as shown for infarct size. Values of infarct volume and brain swelling are shown as a percentage of the contralateral hemisphere in Figure 2.

#### Neuroscore

Group H showed a neuroscore with a median of 2 (range 1 to 4) which was significantly better than group Co with a score of 3 (range 2 to 4) and T with a median of 3.5 (range 3 to 4) (p < 0.05). In addition, group TH had a median score of 2 (range 2 to 4) and therefore performed better than group T (p < 0.05). There were no significant differences detectable between the other groups.

#### Invasion of MPO positive cells

**Figure 3:**
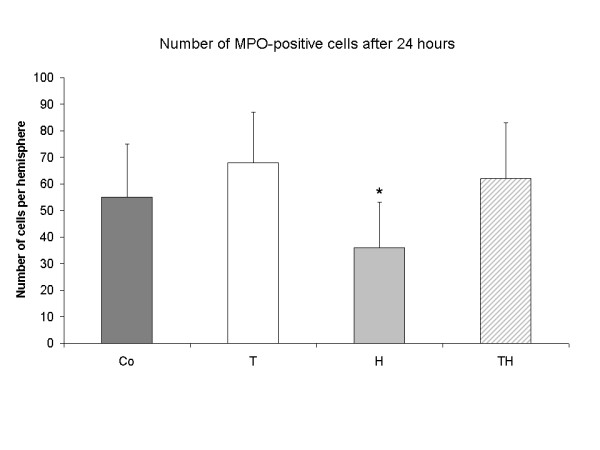
**The number of MPO-positive cells in the infarcted hemisphere is shown after 24 hours**. The group H showed less positive cells compared to all others (p < 0.05) as indicated by the asterisks. There were no other significant differences between the groups.

MPO-positive cells were detectable only in the ischemic hemisphere. The proportion of invaded leukocytes into the infarcted hemisphere in group H was 33% of those counted in the control group and significantly lower compared to all other (p < 0.05). Data of absolute cells count per infracted hemisphere are shown in Figure 3. The number of MPO-positive cells was 55 ± 20 for group Co, 68 ± 19 for group T, 36 ± 17 for group H, and 62 ± 21 for group TH.

#### Serum concentration of sICAM, and TIMP-1

##### sICAM

**Figure 4:**
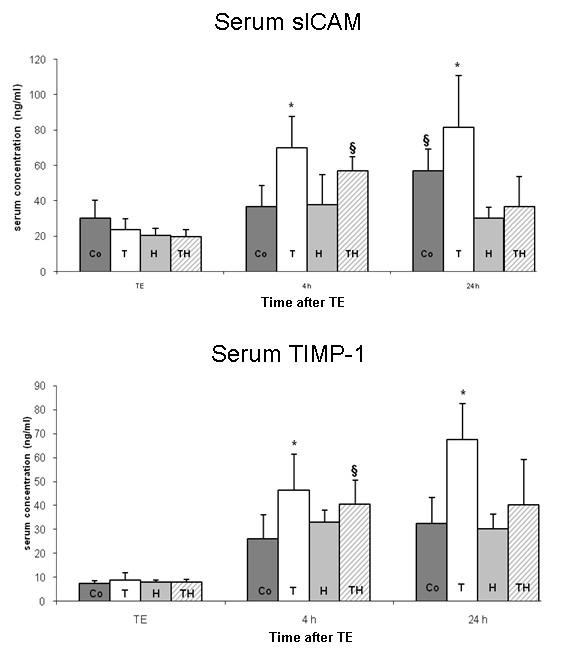
**Serum concentrations of sICAM (top) and TIMP-1 (bottom) are presented as mean ± SD**. SICAM concentration was larger 4 hours after TE in group T compared to group Co and H (p < 0.001; asterisks). SICAM-concentration was larger in group TH compared to group Co and group H (p < 0.05). After 24 hours, sICAM concentration was larger in group T compared to group H and TH (p < 0.01) and group Co (p < 0.05). The concentration in group Co was larger compared to group H and TH (p < 0.01). Levels of significance between the groups were the same for TIMP-1 concentrations after 4 hours. The TIMP-1 concentration after 24 hours was significantly larger compared to group Co and H (p < 0.01) and group TH (p < 0.05) as indicated by the asterisks.

*Levels *in the serum increased over time in group Co and T. In contrast, hypothermia reduced the increase of sICAM-1 serum levels even when combined with rt-PA at different time points. sICAM-levels at 4 hours were larger in group T compared to group Co and H (p < 0.001). After 24 hours, sICAM-levels were lower for group H and TH compared to all other groups (p < 0.05). Data is shown in Figure 4.

##### TIMP-1

Serum-levels showed a similar course as observed for sICAM-1. A time dependent increase was pronounced in group T. Hypothermia reduced increase of TIMP-1 and showed significantly lower levels after 4 and 24 hours compared group T (p < 0.01). Treatment by hypothermia significantly reduced TIMP-1 levels in group TH compared to group T at 24 hours (p < 0.05). Results of TIMP-1 levels are shown in Figure 4.

#### In situ Gelatinase activity

**Figure 5:**
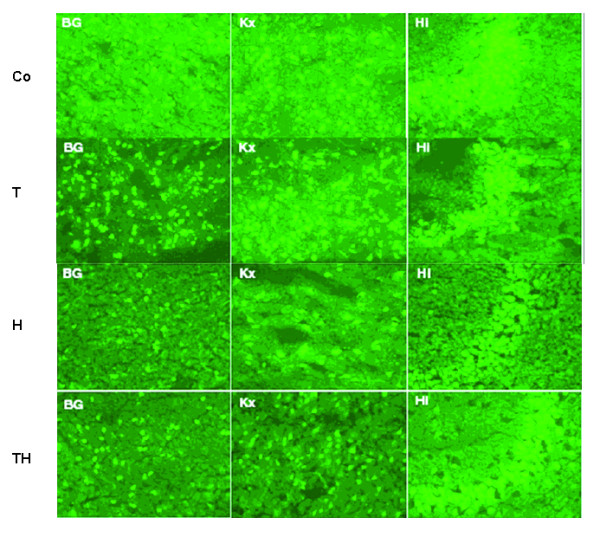
**Exemplary brain slices of gelatinase-activity measurement in different ROIs are shown**. Higher fluorescence intensity could be observed in group Co and group T. Hypothermia reduces gelatinase activity. Reduction of activity is most prominently in the area of the hippocampus. BG:Basal ganglia; Kx: Cortex; Hi: Hippocampus CA1-region. Bar: 50 μM.

**Figure 6:**
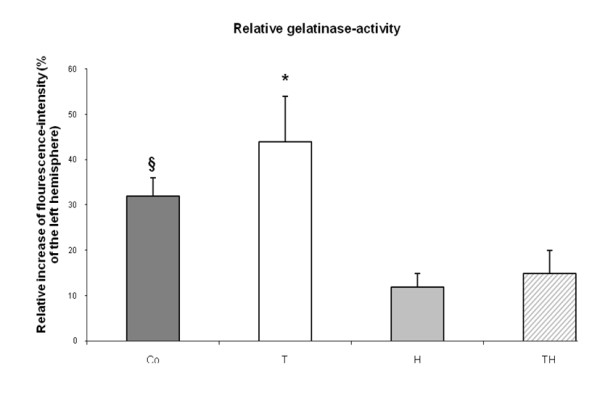
**Relative gelatinase-activity is shown as a percentage of the left, non-ischemic hemisphere and expressed as mean ± SD**. Highest activity could be observed in group T which is significantly higher compared to group Co (p < 0.05) and the groups H and TH (p < 0.01) as indicated by the asterisks. The fluorescence intensity is significantly higher in group Co compared to group H and Th (p < 0.05) as indicated by the string sign.

Gelatinase activity was higher in the ischemic hemisphere compared to the contralateral non-ischemic hemisphere in all sections. The number of cells showing gelatinase activity was superior in parietal cortex compared to the other two investigated regions of basal ganglia and hippocampus (data not shown). Gelatinase activity was reduced in the cortex by hypothermia and combination therapy. Immunohistochemical staining with NeuN indicated predominantly neurons to have gelatinase activity. Quantitative measurement of fluorescence-activity in different ROIs showed a distinct increase in the ischemic hemisphere compared to the contralateral side of the brain. However, hypothermic treatment alone and in combination with rt-PA lead to smaller activity levels compared to the control group Co (p < 0.05) and group T (p < 0.01). RT-PA treatment alone lead to higher activity levels compared to control (p < 0.05). Hypothermia as mono-therapy showed the lowest level of gelatinase activity. Exemplary brain slices are shown in Figure 5 and relative gelatinase activity is shown in Figure 6.

#### Magnetic resonance imaging

##### DWI

There were no significant differences detectable between all treatment groups after 0.5 hours. Exemplary brain slices of DWI are shown in Figure 6 in comparison to T2-WI.

##### T2-WI

**Figure 7:**
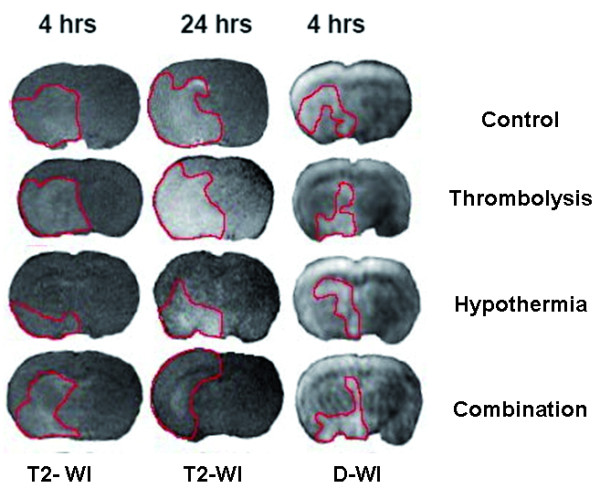
**Exemplary slides of T2-WI and DWI are shown at different time points**. Lesion size is smaller in group H and TH compared to the normothermic groups. Hyperintense and therefore injured regions discriminate as bright contrast from dark background and are pointed out by a red line.

Group H showed smaller infarct size after 4 hours compared to all other groups (p < 0.05). There were no further significant differences comparing all other groups 4 hours after TE. The size of lesion volumes measured by T2-WI after 24 hours was similar to the results observed for histological analysis by the SIS-method and showed same significant differences. Exemplary brain slices of T2-WI and DWI are shown in Figure 7.

##### PWI

Analysis of rrCBV showed a decrease below 25% which was not different between the treatment groups after 0.5 hours in all investigated ROIs.

## Discussion

This study investigated the effects of hypothermia at a level of 34°C body core temperature in a model of thromboembolic stroke and thrombolysis by rt-PA. Hypothermia lead to the smallest infarct size, reduced brain swelling, and improved functional neurologic outcome. This effect was accompanied by reduced serum markers for the disruption of the BBB. Administration of rt-PA 90 min after TE resulted in large infarcts and brain swelling which was associated with high levels of sICAM, TIMP and gelatinase-activity. These effects were diminished by induced hypothermia.

### Stroke model

In contrast to widely used methods with filament middle cerebral artery occlusion (MCAO), a model of thromboembolic stroke was applied in this study. It may reflect the effects of rt-PA, including gradual vessel recanalization and microcirculatory consequences more reliably and offers the opportunity to investigate reperfusion and their effects on the injured brain in a translational approach. Compared to a previous study, in which the injection of red blood clots and rt-PA treatment led to a rather high mortality rate of 40 to 60% [19], this time methodical modifications have achieved a satisfying survival rate (77%) and allowed further investigations after 24 hours in an adequate number of animals. As shown in our preliminary study, we were able to reach this satisfying model by modifying clot size and number. MRI data including DWI 30 min after TE indicated reproducible and similar damage in all animals and perfusion imaging data showed significantly decreased CBF independently from the group. Therefore, stable vessel occlusion without spontaneous reperfusion of the clots can be suggested for the first time period. Since diffusion and T2-weighted imaging showed ischemic brain damage, reproducible infarcts were induced by this model of stroke.

### Effects of thrombolysis on the BBB

Rt-PA treatment after 90 min after TE had two major effects in the present study compared to the control group: both, brain edema were larger and indicators for the disruption of the BBB were increased. S-ICAM-1 is a relevant marker of adhesion. Experimental [23] and clinical studies showed its increase after ischemic stroke [24] and association with increased early mortality [23]. Experimentally, s-ICAM-1 is upregulated after embolic stroke [23]. The integrity of the basal lamina is in major parts influenced by non-cellular proteolytic mechanisms. Matrix metalloproteinases (MMPs) and their endogenous inhibitors, the tissue inhibitors of MMPs (TIMPs) are both mediators in this system and blood values may be a surrogate for the integrity of the BBB [25]. Clinically, reperfusion has been associated to reperfusion injury in a subgroup of stroke patients. In MRI, the so called HARM (hyperintense acute reperfusion marker) indicated disruption of BBB and was associated with hemorrhagic transformation and worse clinical outcome after adjustment for initial severity [26]. Since the timing of the disruption was early enough to be relavant for potential acute thrombolytic therapy, early BBB disruption may be a promising target for adjunctive therapy to reduce the complications associated with thrombolytic therapy, broaden the therapeutic window, and improve clinical outcome.

### Effects of hypothermia

The neuroprotective effects of hypothermia are described in numerous animal experiments of ischemic stroke and include a multitude of mechanisms [26,27]. Hypothermia reduces for example the cerebral metabolism, the release of neurotoxic aminoacids and oxygen free radicals, the apoptotical rate, the invasion of leucocytes and the break-down of the blood-brain-barrier [16,18]. However, the transfer of experimental data on hypothermia for stroke therapy has not been done successfully due to various reasons. First of all, 33°C as the goal temperature used in earlier clinical studies has not -even experimentally - been proven to be superior to any other temperature. A recent study showed a U-shaped curve of efficacy of hypothermia with an advantage for 34°C compared to 33 or 32°C [16]. We used 34°C as the goal temperature for the first time in a model of TE and rt-PA treatment. As seen by the low mortality rate and best neuroscore, this target temperature was well tolerated. Moreover, hypothermia reduced the infarct volume, brain swelling, and invasion of myeloperoxidase cells into the ischemic hemisphere compared to the control group. This is in accordance to data from the filament occlusion model [16]. The reduction of leukocytes in the ischemic hemisphere contributes to a reduction in the release of oxygen species, thrombosis, disruption of the BBB, cerebral edema and plugging of the microvasculature [11,16,18]. Additionally, hypothermia reduced the levels of sICAM-1 and gelatinase activity levels in the brain after 24 hours compared to the control group.

The key question of the recent study was whether hypothermia could influence potentially adverse effects of rt-PA treatment. Although the neuroprotective capacity of hypothermia implicates combination studies with rt-PA, this topic has been investigated by a very limited number of animal studies [19,28]. In a previous study, no additional effects of hypothermia (32°C) could be shown compared to rt-PA treatment alone [28]. In their study, thrombolysis by rt-PA 20 mg/kg lead to a favourable outcome and therefore beneficial effects of hypothermia could have been overseen. Moreover, hypothermia was induced before embolisation which represents a clinically irrelevant scenario. In another study, hypothermia of 33°C started 1 hour after TE [19]. The treatment reduced infarct volume and mortality compared to the control group, but not to any other group after 24 hours. Probably the high mortality rate of the model made it difficult to observe robust effects of combination therapy. In the present study, an advanced TE model with higher survival rates was used. The combination of hypothermia with rt-PA was superior to thrombolysis alone with respect to levels of sICAM, TIMP-1 and gelatinase activity.

## Limitations

Animal models never display the clinical situation 1:1. Intravenous rt-PA is approved for the treatment of acute stroke in a time window from symptom onset of up to 4,5 hours. However, in this model of TE, detrimental effects of rt-PA are recorded already in the 90-minutes time window. Under different experimental settings, such as suture occlusion of the middle cerebral artery, as seen in the especially instructive studies of Nowak and co-workers [29,30], related effects may show different time courses. Another major concern on the combination of hypothermia with rt-PA addresses the theory, that lytic activity could be reduced under lower body temperatures [31] and reperfusion effects might be reduced, therefore. However, the thrombolytic efficacy of rt-PA according to body temperature was not assessed by the present study protocol. Further work should exclusively concentrate on that issue.

## Conclusions

The present study shows that mild hypothermia of 34°C is neuroprotective in a thromboembolic stroke model when focussing on a 24-hour observation time period. Moreover, combination with delayed rt-PA treatment resulted in reduction of infarct volume and parameters indicating a breakdown of the BBB. Therefore, it can be suggested that mild hypothermia can reduce side effects of rt-PA associated treatment. Further investigations need to evaluate whether hypothermia can extend the therapeutic time window for thrombolysis.

## Competing interests

The authors declare that they have no competing interests.

## Authors' contributions

RK designed and performed the experiments, interpreted the data and wrote the manuscript. BK analysed the data and wrote part of the manuscript. SS designed the experiments. All authors approved the final version of the manuscript.
